# Editorial: Pulmonary embolism—New diagnostic and therapeutic strategies

**DOI:** 10.3389/fmed.2023.1281073

**Published:** 2023-11-28

**Authors:** Carlos Jerjes-Sanchez, Mateo Porres-Aguilar

**Affiliations:** ^1^Tecnológico de Monterrey, Escuela de Medicina y Ciencias de la Salud, Instituto de Cardiología y Medicina Vascular, Tecsalud, Monterrey, Mexico; ^2^Division of Adult Thrombosis, Department of Internal Medicine, Hospital Medicine, Texas Tech University Health Sciences Center, El Paso, TX, United States

**Keywords:** venous thromboembolism, pulmonary embolism, deep venous thrombosis, PERT, thrombolysis

Venous thromboembolism (VTE), comprising deep vein thrombosis (DVT) and pulmonary embolism (PE), affects ~10 million people every year worldwide (1). Despite therapeutic advances, the incidence of VTE continues to rise with increased life expectancy and the prevalence of conditions predisposing to VTE (1). VTE remains the first cause of preventable hospital death, associated with short- and long-term morbidity, disability, and healthcare system costs (1). Up to 10 and 25% of patients experience recurrent VTE at 1 and 5 years, respectively, with an associated case fatality rate of 5% (1). Additionally, PE is the third most frequent cause of cardiovascular mortality after ST-elevation myocardial infarction and stroke. It is the leading cause of pregnancy-related maternal death in developed countries (2), the second cause of cancer, and the third in the first 24 h in trauma patients (3).

In the pathogenesis of VTE, multiple factors related to different inflammation grades can interact, and restricted mobility could trigger thromboinflammation and acute VTE events (4). Inflammation could be the pathogenic substrate common to several VTE-predisposing conditions (1). Surgery is a potent proinflammatory stimulus associated with an increased risk of VTE that extends for several months after the procedure (1). Acute and chronic subclinical infections account for a 15-fold increased VTE incidence (1) throughout inmunothrombosis (4). Low-grade intensity, chronic inflammation (obesity, chronic pulmonary obstructive disease (COPD), heart failure, chronic infections, etc.), and high-grade intensity (cancer, major trauma, acute infections, myocardial infarction, chemotherapy, autoimmune disorders, etc.) provide a strong prothrombotic milieu promoting VTE (1).

In this issue, readers will identify the association between PE and SARS-CoV-2, the safety and effectiveness of a third-generation thrombolytic in high-risk PE, the inter-hospital barriers to transferring PE patients, and elements to improve the care of patients with cirrhosis portal vein thrombosis, and hemodynamic and respiratory support in massive PE patients.

In a single-center prospective cohort study (Suarez Castillejo et al.), investigators explored the clinical, radiological, and biological characteristics of 179 consecutive hospitalized patients with COVID-19 pneumonia. Investigators developed the Pulmonary Artery Thrombosis in COVID-19 Mallorca (PATCOM). The overall incidence of pulmonary embolism (PE) was 39.7% (71 patients; CI 95%, 32–47%). In patients with PE, emboli were located mainly in segmental/subsegmental arteries (67%). Pneumonic COVID-19 patients with D-dimer values >1,000 ng/mL were presented with a very high incidence of PE, regardless of clinical suspicion. The authors identified significant differences in urea, D-dimer, platelet distribution width (PDW), neutrophil-to-lymphocyte ratio (NLR), and lymphocyte count between patients with PE and non-PE. The PATCOM score represents a promising PE prediction rule, although validation in further studies is required.

A large, comprehensive meta-analysis and systematic review (Zhang et al.) assessed the efficacy and safety of tenecteplase in patients with acute PE, including six studies, four randomized controlled trials (RCTs), and two cohort studies in patients with high-risk PE. Tenecteplase increased the 30-day survival rate (16 vs. 6%; *p* = 0.005) without a significant increase in the incidence of bleeding (6 vs. 5%; *p* = 0.73). Tenecteplase did not affect short-term nor long-term mortality; however, it was associated with higher bleeding risk (RR = 1.79, 95% CI [1.61–2.00]) in intermediate-risk PE patients. Investigators concluded that tenecteplase is a promising third-generation fibrinolytic drug for patients with high-risk PE and is not recommended for those with intermediate-risk PE.

In an inductive qualitative descriptive analysis performed by DeBerry et al., investigators explored and identified barriers that impede interhospital transfers (IHT) of patients with complex life-threatening acute PE. The most prominent issues identified as barriers to IHT for patients with acute life-threatening PE were (a) inefficient communication, (b) subjectivity in the indication for transfer, (c) delays in data acquisition (imaging or clinical), and (d) operational barriers. Facilitators enabling transfer patients were (a) good communication and (b) a dedicated transfer team. Such themes help identify opportunities to optimize the IHT of patients with acute PE and improve patient care; these opportunities may consider instituting dedicated PE response teams (5).

Portal vein thrombosis (PVT) represents a common hypercoagulable vasculopathy in liver disease. A practical clinician-oriented comprehensive review (Prakash et al.) was well-written to enhance the knowledge of managing cirrhotic PVT, as it may help to reduce portal hypertensive complications. An individualized assessment of risks vs. benefits is necessary when deciding between different treatment strategies, including the current role of oral factor Xa inhibitors.

Lastly, Pérez-Nieto et al. performed a practical narrative review, critically discussing and summarizing hemodynamic and respiratory support strategies in PE, including vasopressors, inotropes and vasodilator therapies, oxygen therapy, ventilation, mechanical circulatory support including VA-ECMO and right ventricular assisted devices. Such recommendations are mainly based on low-quality levels of evidence (case series, retrospective observational data, etc.). Investigators firmly concluded that high-quality research data is needed to inform clinical decision-making in severe acute PE better.

## Author contributions

CJ-S: Writing – original draft, Writing – review & editing. MP-A: Writing – review & editing.
